# Heterotypic Cell Culture from Mouse Bone Marrow under Simulated Microgravity: Lessons for Stromal Lineage Functions

**DOI:** 10.3390/ijms241813746

**Published:** 2023-09-06

**Authors:** Elena Markina, Ekaterina Tyrina, Andrey Ratushnyy, Elena Andreeva, Ludmila Buravkova

**Affiliations:** Cell Physiology Laboratory, Institute of Biomedical Problems, Russian Academy of Sciences, 123007 Moscow, Russia; eagolikovamsu@gmail.com (E.T.); ratushkin@mail.ru (A.R.); buravkova@imbp.ru (L.B.)

**Keywords:** microgravity simulation, 3D clinorotation, mice, bone marrow, stromal lineage cells, multipotent mesenchymal stromal cells, extracellular matrix, transcriptomics

## Abstract

Muscle and skeleton structures are considered most susceptible to negative factors of spaceflights, namely microgravity. Three-dimensional clinorotation is a ground-based simulation of microgravity. It provides an opportunity to elucidate the effects of microgravity at the cellular level. The extracellular matrix (ECM) content, transcriptional profiles of genes encoding ECM and remodelling molecules, and secretory profiles were investigated in a heterotypic primary culture of bone marrow cells after 14 days of 3D clinorotation. Simulated microgravity negatively affected stromal lineage cells, responsible for bone tissue formation. This was evidenced by the reduced ECM volume and stromal cell numbers, including multipotent mesenchymal stromal cells (MSCs). ECM genes encoding proteins responsible for matrix stiffness and cell-ECM contacts were downregulated. In a heterotypic population of bone marrow cells, the upregulation of genes encoding ECM degrading molecules and the formation of a paracrine profile that can stimulate ECM degradation, may be mechanisms of osteodegenerative events that develop in real spaceflight.

## 1. Introduction

Microgravity is one of the most important factors determining the state of living beings in spaceflight. In studying the mechanisms of microgravity effects, the analysis of bone tissue remodelling is of particular importance. Atrophic changes in bone tissue have been observed in astronauts after long-term missions [1,2,3,4,5,6,7,8]. In general, there was a loss of 1.0–1.5% of bone mineral density per month of spaceflight [2].

Experiments with animals in space suggested that the impairment of bone formation could be considered a key event in bone loss. For instance, bone formation was suppressed during spaceflight in young rats. In mature rats, bone resorption predominated with little change in bone formation [9,10,11,12,13,14,15]. It was suggested that the observed changes in bone tissue may be related to the disruption of extracellular matrix (ECM) remodelling in the cells of the stromal lineage [16,17]. Various techniques for ground-based simulation of microgravity effects provide an opportunity to at least partially fill this gap. Several approaches have been developed to simulate the effects of spaceflight in ground-based conditions. These include physiological level or in vitro experiments. To study the effects of microgravity on cells, various devices have been developed to simulate gravity deprivation. Currently, 3D clinorotation, which provides continuous random changes in orientation relative to the vector of gravity, is most widely used for studies at the cellular level [18].

Exposure to microgravity for several days showed a marked suppression of differentiation of human bone marrow mesenchymal stem cells into osteoblasts, manifested by decreased expression of *ALP*, a lack of mineralization, and downregulation of *COL1A1*, *SPARC,* and *RunX2* [19]. A change in the functional activity of bone marrow stromal progenitors led to remodelling of the ECM. The genes encoding collagen types I, III, IV, V, VI, and VIII; osteocalcin; and osteopontin were downregulated, whereas matrix metalloprotease genes were upregulated [20,21,22,23,24,25].

The above data suggest that the metabolism of the ECM of stromal lineage cells is seriously compromised following microgravity. It is known that the renewal of the bone osteogenic cells comes from the pool of stromal lineage cells localized in the bone marrow, in which they are a part of the haematopoietic niche. Haematopoietic lineage cells have a strong paracrine effect on the functional activity of stromal cells [26]. Therefore, to study the effects of simulated microgravity on the state of the stromal lineage cells, we used a heterotypic population of adherent cells from mouse bone marrow. This was performed to preserve the close interaction with the rest of the bone marrow cells that is so important for the fate of osteoprogenitors.

In this work, the ECM content, the transcriptional profiles of genes encoding ECM and remodelling molecules, and the secretory profiles of cultured cells were investigated in a heterotypic primary cell culture of bone marrow cells.

## 2. Results and Discussion

### 2.1. Characterization of the Heterocellular Bone Marrow Population in Primary Culture

Marrow-derived adherent cells are a well-established heterocellular population that comprise at least two morphological subtypes: fibroblastic colonies and clusters of macrophages [27,28]. They have long been used in vitro experiments to study the effects of various factors on haematopoietic cells, including those from bone marrow [27,28]. We applied this cellular model to study the effects of simulated microgravity on stromal lineage cells in heterotypic cooperation.

**Figure 1:**
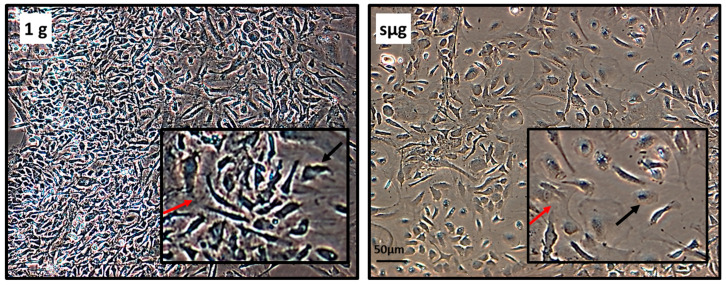
Bone marrow cells after 14 days in primary culture. Representative images, phase contrast, magn. 200×. Higher magnification in insets: stromal fibroblast-like cells are marked with a red arrow; macrophage-like cells are marked with a black arrow.

After 14 days in culture, differences in cell morphology were observed between Earth’s gravity (1 g) and simulated microgravity (sμg) samples (Figure 1). In the 1 g group, there were numerous areas of high cell density corresponding to the morphological features of bone marrow stromal cells. Macrophage-like cells were located at the periphery of such clusters. After sμg, there were fewer such cell clusters and the density of cells within them was lower. Macrophage-like cells were present in greater numbers and were located in the space between the stromal cells (Figure 1).

After enzymatic detachment, cells were counted. Adherent cell numbers in the 1 g group were 1.4 × 10^6^ ± 0.04 (*p* ≤ 0.05) compared to 1.1 × 10^6^ ± 0.04 (*p* ≤ 0.05) following sμg. Thus, there was a small but significant decrease in total cell numbers after microgravity simulation.

**Figure 2:**
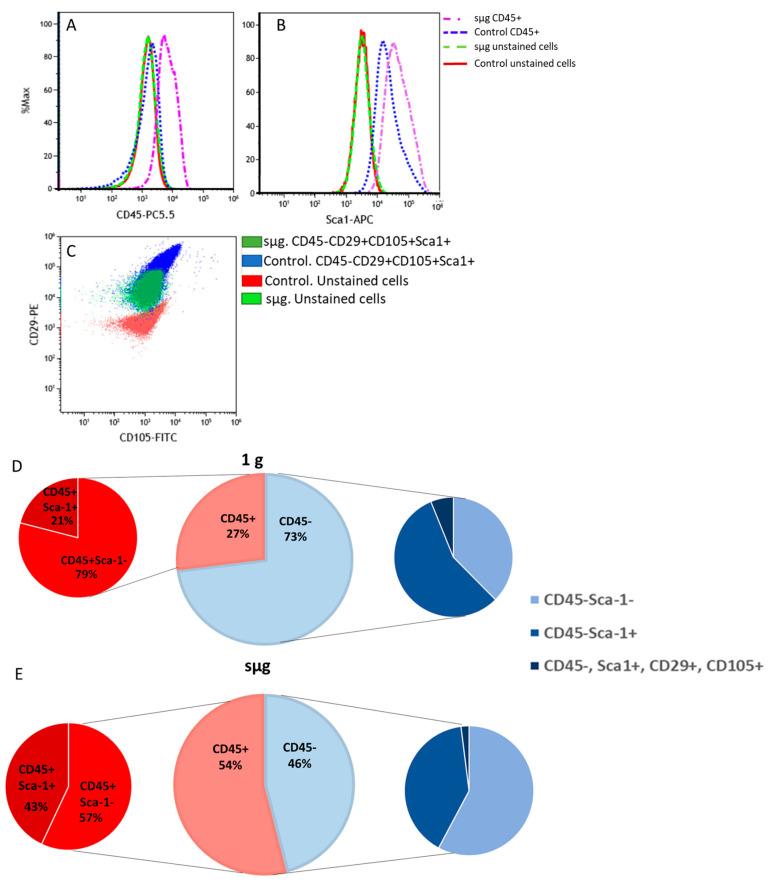
Flow cytometry analysis of heterotypic cell populations from mouse bone marrow. (**A**) Cells were gated based on CD45. (**B**) Cells were gated based on Sca-1. (**C**) Four-color immunophenotyping of murine bone marrow cells using combinations of PE (CD29), FITC (CD105), PerCP (CD45), and APC (Sca-1). Representative dot plots show original compensated data (logarithmic display). (**D**) Percentage of cells with different phenotypes, 1 g; (**E**) percentage of cells with different phenotypes, sμg.

Phenotyping of cultured bone marrow cells was performed using cytofluorometric analysis. A panel of antibodies against the following antigens was used: CD29, CD45, CD105, and Sca-1 (stem cell antigen). This allowed for the separation of hematopoietic (CD45+) and stromal (CD45−) cells (Figure 2A). The presence of Sca-1 was used to determine the proportion of progenitor cells (Sca-1+) in both populations (Figure 2B). In addition, among the stromal cells, we determined the proportion of multipotent mesenchymal stromal progenitor cells (MMSCs) characterized by their phenotype (CD45−, CD29+, CD105+, and Sca1+) (Figure 2C). Three-quarters of the cells after 1 g belonged to the stromal differon (CD45−) (Figure 2D). Two-thirds of the latter cells could be attributed to stromal precursors (CD45−Sca1+) (Figure 2D). 

After microgravity simulation, the proportion of stromal cells (CD45−) decreased and represented about half of all bone marrow cells (Figure 2E). Approximately one-third of the stromal cells had a progenitor phenotype (CD45−Sca1+) (Figure 2E). The percentage of hematopoietic progenitors (CD45+Sca1+) among the CD45+ after sμg was 43%, while following 1 g, it was approximately 10% (Figure 2D,E). The percentage of MSCs (CD45−, CD29+, CD105+, and Sca1+) in 1 g was 11% (Figure 2D). In sμg, the percentage of MSCs was half as high (Figure 2E).

Thus, exposure of the mouse bone marrow heterocellular population to simulated microgravity resulted in a decrease in the total number of cells. Morphological analysis, using flow cytometry, revealed a change in the ratio of stromal to hematopoietic cells in favour of the latter. Not only did the total number of stromal cells decrease at sμg but also stromal precursor cell numbers were reduced, including the most primitive—MSCs.

A decrease in the proportion of stromal lineage cells could be caused by a reduction in their proliferative activity. Such an effect has previously been described after microgravity simulation. Clinorotation of human MSCs for 1 h to 10 days and for 20 days resulted in inhibition of their proliferation [29,30]. Three-dimensional clinorotation of rat bone marrow mesenchymal cells for 24–96 h also resulted in arrest in the G0/G1 phase and inhibition of proliferation [31].

### 2.2. Stromal Lineage Cells and Extracellular Matrix

It is reasonable to suppose that if the proliferative activity of stromal cells is significantly impaired, other functions may also be compromised, particularly the major stromal function of extracellular matrix (ECM) production. The ECM of mammalian tissues in vivo is a complex structure consisting of many molecular components such as fibrils, fibril-associated cross-linking elements, and specific ligands that interact with cellular receptors [32]. Physical aspects include stiffness, elasticity, viscoelasticity, etc. [33].

**Figure 3:**
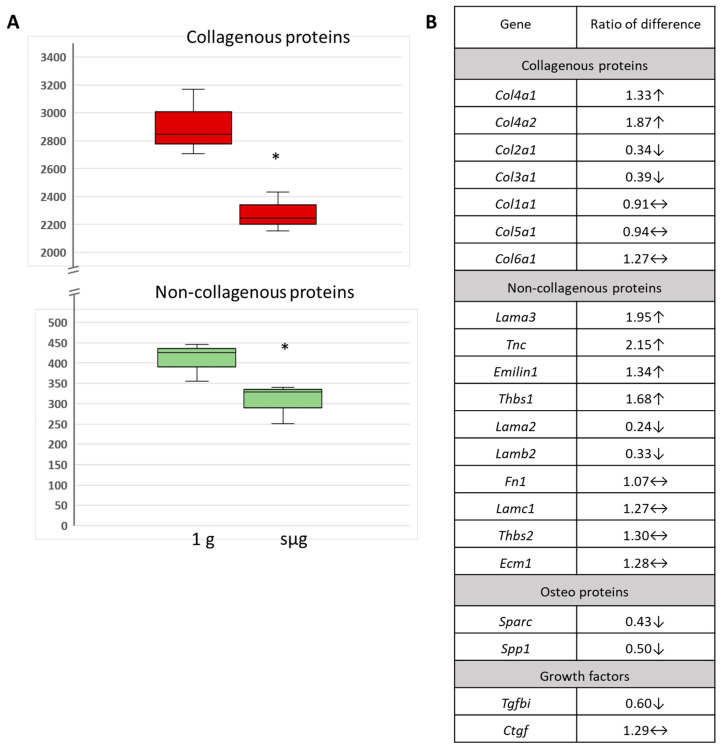
The effects of simulated microgravity on the ECM content and transcriptomic activity of genes encoding ECM proteins in cultured bone marrow cells. (**A**) Semiquantative colorimetric estimation of collagenous and non-collagenous proteins. * Significant difference from 1 g. Data are presented as mean ± SEM (M ± SEM), *p* ≤ 0.05. (**B**) Differential expression of genes encoding structural components of ECM in primary bone marrow cells. The data are presented as fold changes, sμg vs. 1 g. ↑—up; ↓—down; and ↔—no change.

After exposure at 1 g and sμg, ECM proteins were detected histochemically in bone marrow cell cultures (Figure 3A). According to a semiquantitative colorimetric assay, the amounts of both collagenous and non-collagenous proteins were decreased following sμg (*p* ≤ 0.05) (Figure 3A). Earlier studies have shown similar changes in collagenous and non-collagenous protein levels after 10 days of 3D clinorotation of MSCs from human adipose tissue [34]. In addition, a decrease in alkaline phosphatase activity and ECM in rat bone marrow MSCs and human MSCs was observed after 14 days of 3D clinorotation [19,35,36].

We hypothesized that the detected changes in ECM components following microgravity simulation might be associated with changes in the transcriptional activity of the corresponding genes. To analyse the differential gene expression, we used the RT^2^ Profiler PCR Array Mouse Extracellular & Adhesion Molecules Kit, which allows us to evaluate the activity of genes encoding different functional groups of the ECM and its associated molecules (Figure 3B). After sμg, the expression profile of ECM genes changed. The genes encoding bone interstitial fibrillar collagen types II and III were downregulated (Figure 3B). *Tnc, Emilin1,* and *Thbs1* encoding protein cores of corresponding glycoproteins demonstrated elevated transcription. Changes in the transcription of the major components of the basement membrane were also observed. Collagen IV (*Col4a1* and *Col4a2*) mRNA increased. Downregulation of the genes encoding laminin-221 (*Lamb2* and *Lama2*) was detected. Laminin-221 belongs to the classical laminin subfamily [37], typical of stiff tissues [38]. The gene for laminin-321 (*Lama3*) (naked laminin subfamily), typical of skin, was upregulated. At the same time, the transcription of the fibronectin gene, whose protein ensures the binding of laminins to collagen IV in the basement membrane, did not change (https://reactome.org/PathwayBrowser/, accessed on 19 May 2023).

Previously, downregulation *of COL1A* and *COL1A2* has been reported in human bone marrow MSCs and mouse osteoblasts after microgravity simulation in vitro for 3–21 days [19,39,40,41,42,43]. Microgravity exposure of human fibroblasts also previously resulted in upregulation in COL4A5 [44], in agreement with our study.

As shown previously, ECM stiffness can determine the functional activity of cells. For example, proliferation and osteodifferentiation of bone marrow MSCs are more pronounced on stiffer substrates [45,46]. Downregulation of collagen II and III expression in sμg culture resulting in a reduction in ECM stiffness may also influence the expression of other matrix proteins. If this is the case, proliferative activity and osteodifferentiation may be reduced after microgravity simulation. Indeed, similar effects have been observed in human bone marrow MSCs after 14 days of 3D clinorotation [47]. The changes in the ratio of stromal progenitors with different levels of commitment at sμg described above could be explained if a similar pattern was realized in our experiments. That is, a decrease in the total number of stromal cells and a decrease in the number of stromal precursors, including MSCs.

### 2.3. Transcriptomic Analysis of Genes Encoding Regulatory Molecules

**Figure 4:**
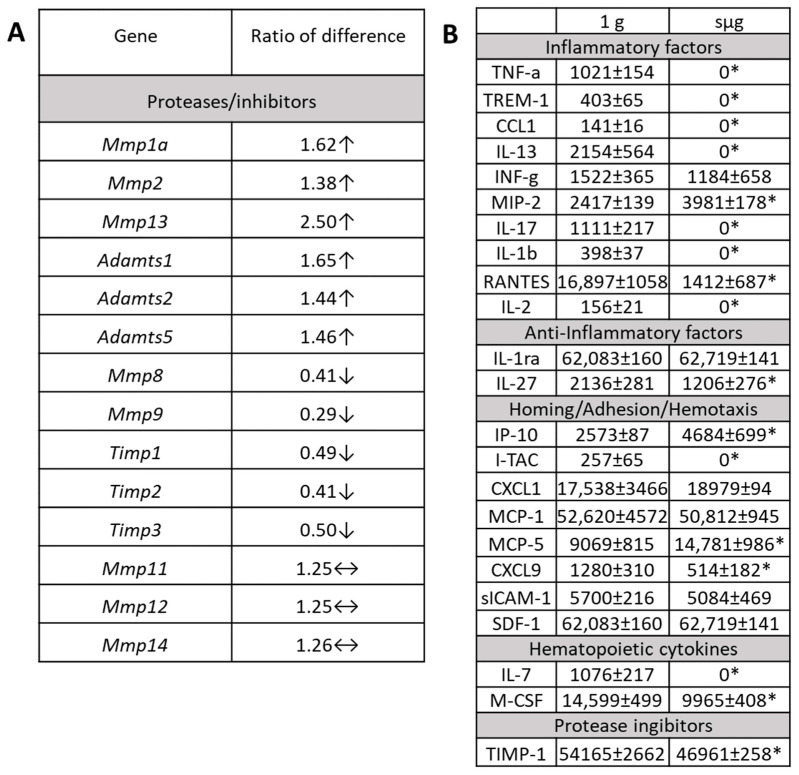
The effects of simulated microgravity on transcriptomic and secretory profiles of cultured bone marrow cells. (**A**) Differential expression of genes encoding matrix metalloproteases and their tissue inhibitors. The data are presented as fold changes, sμg vs. 1 g. ↑—up; ↓—down; and ↔—no change. *Mmp* (*1a*, *2*, *8*, *9*, *11*, *12*, *13*, and *14*)—matrix metalloproteinase (1a, 2, 8, 9, 11, 12, 13, and 14); *Adamts* (*1*, *2*, and *5*)—a disintegrin and metalloproteinase with thrombospondin motifs (1, 2, and 5); *TIMP* (*1*, *2*, and *3*)—tissue inhibitors of metalloproteinases (1, 2, and 3); and (**B**)—paracrine mediator levels in conditioned medium of bone marrow cultures. The levels of cytokines were characterized semiquantitatively using a fluorescent dot-blot assay. The data are presented as arbitrary units of fluorescence. *—significant difference from 1 g. Data are presented as M ± SEM, *p* ≤ 0.05.

The above results from the analysis of genes encoding structural proteins of the ECM are mostly related to stromal lineage cells, since they are the predominant contributors to ECM production in bone marrow. At the same time, the source of paracrine factors that govern ECM remodelling is not only the stromal cells themselves. It is also, perhaps to a greater extent, the hematopoietic cells that are present in large numbers in the bone marrow. Therefore, the data on changes in the transcriptional activity of genes of those molecules involved in ECM remodelling, as well as the paracrine profile in heterocultures, certainly apply to cells of both stromal and hematopoietic lineages (Figure 4A).

The estimation of MMP activity has several technical limitations. In particular, MMPs are expressed as zymogens that require activation in tissue via various mechanisms before they can have a biological effect. Therefore, Hardy et al. [48] suggested considering MMP transcription as a likely proxy to characterize MMP activity. We have applied this approach in our experiments. After sμg, an upregulation of MMP genes was observed (*Adamts1*, *Adamts5*, *Mmp11*, *Mmp12*, *Mmp13*, *Mmp14*, *Mmp1a*, and *Mmp2*), while genes encoding MMP inhibitors were downregulated (Timp1, Timp2, and Timp3). The level of Timp-1 in the conditioned medium was also reduced.

It is known that MMP-1, -2, -13, and -14 play important roles in the remodelling of tissue ECM [49]. MMP-1 and -2 degrade collagen I and II [48]. MMP-2, -12, and -14 cleave non-collagenous proteins bound to the bone matrix, such as osteonectin, vitronectin, osteopontin, and bone sialoprotein, and control the availability of growth factors attached to cell membranes and ECM [48]. MMP-2, -12, -13, and -14 play important roles in bone matrix reorganization and regulation of cell–matrix and cell–cell interactions. The reduced activity or absence of MMP-9 is often associated with abnormal bone morphology, in particular impaired ossification and cartilage hypertrophy in tubular bones [50,51,52]. In addition, MMP-14 promotes the release of RANKL and orients the RANK/RANKL/osteoprotegerin axis toward osteoclast maturation and activation. Our data on differential expression of protease/inhibitor genes could suppose the shift in remodelling molecules to enhance ECM degradation [53]. 

Table 1 summarizes the biological processes in which the molecules encoded by the differentially expressed genes are involved. After sμg, the expression of genes whose products are involved in the processes of cell adhesion, cell differentiation, positive regulation of cell communication, regulation of cell migration, extracellular matrix organization, and negative regulation of metallopeptidase activity was downregulated. The upregulated genes encode molecules involved in negative regulation of cell-matrix adhesion, negative regulation of cell adhesion, negative regulation of angiogenesis, cell migration, extracellular matrix degradation, collagen catabolism, and basement membrane organization.

Based on the data presented above, it could be concluded that there was a shift in the transcriptional profile of genes encoding components of the ECM and its remodelling molecules after microgravity simulation. This was manifested via a downregulation of genes encoding fibrillar collagens, whereas genes encoding basement membrane collagens, laminins, and glycoproteins were upregulated. Genes whose products are responsible for catabolic processes in the ECM were also upregulated. In tissues, the activity of such molecules could ensure the predominance of matrix degradation.

After sμg, there was a significant change in the profile of soluble mediators in the conditioned medium of the cultured bone marrow cells (Figure 4B). TNF-a, TREM-1, CCL1, IL-13, IL-2 and IL-7 were not detected. The levels of CXCL9/MIG, RANTES, and IL-27, which regulate macrophage chemotaxis, and macrophage colony-stimulating factor (M-CSF), which determines the differentiation of macrophages [54,55,56,57], were reduced. At the same time, the levels of IP-10, MIP-2, MCP-5 and IL-23 increased. These cytokines are shown to play an important role in the activation of neutrophils and macrophages, including osteoclasts [58,59,60,61,62].

The data on the shift in the profile of soluble mediators led to the conclusion that the paracrine microenvironment created at sμg can stimulate ECM degradation. This can be supported by an increase in IL-23. IL-23 initiates the processes of ECM degradation by stimulating the production of RANKL [63]. At the same time, there was a decrease in the levels of IL-2 and IL-27, which support the production of ECM. Increased levels of the chemokines IP-10, MCP-5, and MIP-2, which enhance macrophage chemotaxis and activation [59,64,65], possibly indicate an increase in cells with proteolytic activity. The secretion of proinflammatory cytokines increased, which may lead to osteoclast activation, which, if TIMP-1 secretion is suppressed, may be a prerequisite for the activation of the ECM degradation processes. This hypothesis is supported by the observed decrease in ECM proteins and in the activity of the genes that encode them. The reduction in regulatory molecules of migration and chemotaxis in the conditioned medium may explain the observed transcriptomic changes of related molecules that govern these processes.

We were therefore able to show that simulated microgravity negatively affects stromal lineage cells, which are responsible for bone tissue formation. This was evidenced by the reduced ECM volume and stromal cell numbers, including MSCs. There was a change in the expression of ECM genes, with a downregulation of genes whose proteins are responsible for matrix stiffness and cell-ECM contacts. There was also increased activity of MMP genes and the formation of a paracrine profile that can stimulate ECM degradation in a heterotypic population of bone marrow cells.

It is generally accepted that microgravity leads to impaired mechanosensitivity and subsequent mechanotransduction [66,67,68]. A complex of extracellular (ECM) and intracellular (cytoskeleton) structures connected by transmembrane proteins is responsible for the perception of mechanical (gravitational) stimuli. The removal of the gravitational load provokes a change in cell mechanotransduction, primarily at the transcriptional level. We assume that the downregulation of genes whose proteins are responsible for matrix stiffness and cell–cell contacts detected in our study is indicative of an impaired mechanotransduction, since most of these proteins are involved in the mechanosensitivity path-way. This, in turn, may lead to the inhibition of cell adhesion and intercellular interaction, as well as stromal lineage cells’ differentiation. On the other hand, we showed that simulated microgravity resulted in increased MMP gene activity and a paracrine profile capable of promoting ECM degradation in a heterotypic bone marrow cell population. We hypothesize that the complex of these gravity-dependent changes may be one of the mechanisms of osteodegenerative phenomena that develop under real spaceflight conditions.

## 3. Materials and Methods

### 3.1. Experimental Animals

We used 6–8-week-old male BalbC mice in this study and followed protocols approved by the Commission on Biomedical Ethics of the Institute of Biomedical Problems (Minutes No. 497 dated 7 July 2018). All mice were housed in cages in the animal facility for at least 1 week for acclimation prior to experimental use. We used cervical dislocation to euthanize the animals.

### 3.2. Bone Marrow Cell Isolation and Enumeration

Right and left femur and tibia bones were obtained from each animal (n = 6). Bone marrow (BM) was isolated according to our modification of the generally accepted procedure [69]. Briefly, BM cells were flushed out from the tibia and femur bone cavities into 50 mL centrifuge tubes. After centrifugation at 500× *g* for 5 min, supernatants were removed and cell pellets were resuspended in full culture medium (a-MEM; Gibco, Life Technology Ltd., Paisley, UK), containing 20% EFV (HyClone laboratories, Logan, UT, USA), 2 mM L-glutamine (Gibco, Life Technology Ltd., Paisley, UK), and streptomycin/penicillin (100 µg/100 units/mL) (PanEco, Moscow, Russia). The viable nucleated cells were counted in a Neubauer chamber. The trypan blue exclusion test was applied to exclude dead cells.

### 3.3. Cell Culture

**Figure 5:**
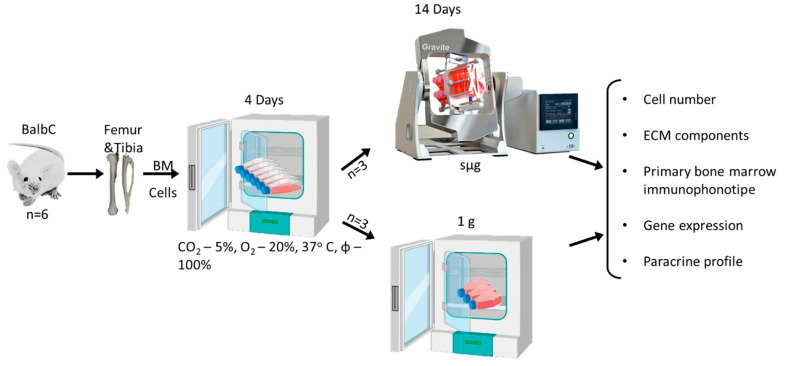
Study design. Bone marrow cells were isolated from hindlimb bones of male BalbC mice (n = 6). After isolation and counting, cells were pooled and divided into 2 groups: 1 g—standard culture conditions and sμg—3D clinorotation during 14 days. There were three independent experiments with two technical repetitions in each group. Cells were seeded in culture flasks for 4 days following 1 g. On day 5, the medium containing non-adherent cells was discarded and the vials were completely filled with fresh medium. Heterotypic bone marrow cell cultures were exposed under 1 g or sμg for 14 days. After the end of the experiment, the cells were used for ex vivo assays.

After isolation and counting, cells were pooled and divided into 2 groups: (1) 1 g—cells following Earth’s gravity (standard culture conditions)—and (2) sμg—cells following 3D clinorotation during 14 days (simulated microgravity). There were three independent experiments with two technical repetitions in each group. Cells were seeded at a density of 6 × 10^5^ cells/cm^2^ in culture flasks (25 cm^2^, NUNC, Nalge Nunc International, Roskilde, Denmark) or slide flasks (10 cm^2^ NUNC, Nalge Nunc International, Roskilde, Denmark) in three replicates. Cultivation was performed in growth medium (a-MEM; (Gibco, Life Technology Ltd., Paisley, UK), containing 20% FBS (HyClone laboratories, Logan, UT, USA), 2 mM L-glutamine (Gibco, Life Technology Ltd., Paisley, UK), and streptomycin/penicillin (100 µg/100 units/mL) (PanEco, Moscow, Russia) for 4 days following 1 g. On day 5, the medium containing non-adherent cells was discarded, the vials were completely filled with fresh medium, air bubbles were removed, and the caps were tightly closed (Figure 5).

### 3.4. In Vitro Simulation of Microgravity Effects (3D Clinorotation)

In our experiments we used Gravite^®^ equipment (Space Bio-Laboratories Co., Ltd., Hiroshima, Japan). According to the manufacturer’s manual, Gravite^®^ is a multidirectional G-force generator controlling the rotation of two axes simultaneously. This unique feature allows for cancellation of the cumulative gravitational vector at the center of the device to create 10^–3^ g. Gravite equipment with culture flasks was placed in the laboratory thermostat (KIM, Kasimov, Russia) at 37 °C. For 1 g, culture flasks were placed on the bottom of the same thermostat. Heterotypic bone marrow cell cultures were exposed to 1 g or sμg for 14 days.

### 3.5. Histochemical Evaluation of ECM Components

Cultured adherent bone marrow cells were fixed with 4% paraformaldehyde and stained with Sirius Red (Thermo Scientific Chemicals, Waltham, MA, USA) to detect collagenous proteins or with Fast Green FCF (Thermo Scientific Chemicals, Waltham, MA, USA) for non-collagenous proteins. The dyes were extracted with 25 mM NaOH in methanol and the optical density of the solution was measured using a PR2100 spectrophotometer (Bio-Rad Laboratories, Hercules, CA, USA).

### 3.6. Immunophenotyping of Cultured Primary Bone Marrow Cells

After 14 days of exposure at 1 g or sμg, cultured adherent bone marrow cells were washed with phosphate-buffered saline (PBS). Trypsin-EDTA solution (Gibco, UK) was used to detach cells. After centrifugation with PBS, cell pellets were resuspended in PBS and used for flow cytometry. A commercial Mouse Mesenchymal Stem Cell Multi-color Flow Cytometry Kit (R&D Systems Inc., Minneapolis, MN, USA) was used for immunophenotyping according to the manufacturer’s instructions. Isotypic IgG conjugated with PE, FITC, PerCP, and APC, included in the kit, was used as a negative control. Cells were analysed using a Cytoflex flow cytometer (Beckman Coulter, Brea, CA, USA) with the following parameters: FITC excitation 488, emission 525/40; PE excitation 488, emission 585/42; PerCP excitation 488, emission 690/50; and APC excitation 638, emission 660/0. The data were analysed using Kaluza Analysis 2.1 data processing and imaging software (Beckman Coulter, Brea, CA, USA).

### 3.7. Quantitative Real-Time PCR

Total RNA was isolated using QIAzol lysis reagent (Qiagen Sciences LLC, Germantown, MD, USA), followed by a reverse transcription reaction using a QuantiTect Reverse Transcription Kit (Qiagen Sciences LLC, Germantown, ML, USA) according to the manufacturer’s instructions. The cDNA was used for quantitative PCR using a commercial RT^2^ Real Time SYBR Green/ROX PCR master mix (Qiagen Sciences LLC, Germantown, MD 20874, USA) and RT^2^ Profiler PCR Array Mouse Extracellular & Adhesion Molecules kit (Qiagen Sciences LLC, Germantown, MD, USA). The expression level was assessed using the 2^−∆∆Ct^ method according to the manufacturer’s recommendation (Qiagen Sciences LLC, Germantown, MD, USA).

### 3.8. Paracrine Activity

Soluble bioactive mediators in conditioned medium from cultured bone marrow cells were measured using a Mouse Cytokine Array Panel A (R&D Systems Inc., Minneapolis, MN, USA) according to the manufacturer’s instructions. After performing dot-blots, the levels of cytokines were characterized by the intensity of chemiluminescence using Bio-Rad ChemiDoc™ Imaging Systems (Bio-Rad Laboratories, Hercules, CA, USA). Image analysis was performed using Image Lab 5.0 software (Bio-Rad Laboratories, Hercules, CA, USA).

### 3.9. Statistical Analysis

Statistical analysis was performed using Microsoft Excel 2010 and Statistica 7.0, using the analysis of variance method. Data are presented as mean ± SEM. The differences were considered significant at *p* < 0.05.

## Figures and Tables

**Table 1:** Gene ontology (GO) enrichment analysis of gene expression data.

Downregulation	Upregulation
Cell adhesion	Negative regulation of cell-matrix adhesion
*Lama2, Lamb2, Tgfbi, Col3a1, Sparc*	*Thbs1*
Cell differentiation	Negative regulation of cell adhesion
*Lama2, Lamb2, Tgfbi, Col2a1, Col3a1, Mmp8, Mmp9, Lama2, Lamb2, Spp1, Timp2*	*Thbs1, Mmp2*
Positive regulation of cell communication	Negative regulation of angiogenesis
*Lama2, Col3a1, Spp1, Timp2, Timp3, Mmp8, Mmp9*	*Adamts1, Adamts5, Col4a2, Thbs1, Emilin1*
Regulation of cell migration	Cell migration
*Lama2, Col3a1, Sparc, Mmp9, Timp1*	*Mmp2, Lama3, Thbs1, Emilin1*
Extracellular matrix organization	Extracellular matrix disassembly
*Tgfbi, Col2a1, Col3a1, Mmp8, Mmp9, Lamb2*	*Mmp13*
Negative regulation of metallopeptidase activity	Collagen catabolic process
*Timp1, Timp2, Timp3*	*Adamts2, Mmp1a, Mmp2, Mmp13*
	Basement membrane organization
	*Col4a1, Col4a2, Lama3*

Data were created using string-db.org. *Mmp* (*1a*, *2*, *8*, *9*, *11*, *12*, and *13*)—matrix metalloproteinase (1a, 2, 8, 9, 11, 12, and 13); *Adamts* (*1*, *2*, and *5*)—a disintegrin and metalloproteinase with thrombospondin motifs (1, 2, and 5); *TIMP* (*1*, *2*, and *3*)—tissue inhibitors of metalloproteinases (1, 2, and 3); *Col* (*2a1*, *3a1*, *4a1*, and *4a2*)—collagen (2a1, 3a1, 4a1, and 4a2); *Lam* (*a2*, *a3*, and *b3*)—laminin (a2, a3, and b3); *Tgfbi*—transforming growth factor, beta-induced; *Sparc*—osteonectin (secreted protein acidic and rich in cysteine); *Spp1*—osteopontin (secreted phosphoprotein 1); *Thbs1*—thrombospondin 1; *Emilin1*—elastin microfibril interfacer 1.

## Data Availability

The data presented in this study are available on request from the corresponding author. The data are not publicly available due to Institution policy.
